# Perioperative CTEPH patient monitoring with 2D phase-contrast MRI reflects clinical, cardiac and pulmonary perfusion changes after pulmonary endarterectomy

**DOI:** 10.1371/journal.pone.0238171

**Published:** 2020-09-14

**Authors:** Christoph P. Czerner, Christian Schoenfeld, Serghei Cebotari, Julius Renne, Till F. Kaireit, Hinrich B. Winther, Gesa H. Pöhler, Karen M. Olsson, Marius M. Hoeper, Frank Wacker, Jens Vogel-Claussen

**Affiliations:** 1 Institute for Diagnostic and Interventional Radiology, Hannover Medical School, Hannover, Germany; 2 Biomedical Research in Endstage and Obstructive Lung Disease Hannover (BREATH), Member of the German Center for Lung Research, Hannover, Germany; 3 Department of Cardiothoracic, Transplantation and Vascular Surgery, Hannover Medical School, Hannover, Germany; 4 Clinic for Pneumology, Hannover Medical School, Hannover, Germany; Medizinische Universitat Graz, AUSTRIA

## Abstract

Magnetic resonance imaging (MRI) is an emerging tool for diagnosis and treatment monitoring of chronic thromboembolic pulmonary hypertension (CTEPH). The current study aims to identify central pulmonary arterial hemodynamic parameters that reflect clinical, cardiac and pulmonary changes after PEA. 31 CTEPH patients, who underwent PEA and received pre- and postoperative MRI, were analyzed retrospectively. Central pulmonary arterial blood flow, lung perfusion and right heart function data were derived from MRI. Mean pulmonary arterial pressure (mPAP) and 5-month follow-up six-minute walk-distance (6MWD) were assessed. After PEA, mPAP decreased significantly and patients achieved a higher 6MWD. Central pulmonary arterial blood flow velocities, pulmonary blood flow (PBF) and right ventricular function increased significantly. Two-dimensional (2D) phase-contrast (PC) MRI-derived average mean velocity, maximum mean velocity and deceleration volume changes after PEA correlated with changes of 6MWD and right heart ejection fraction (RVEF). Deceleration volume is a novel 2D PC MRI parameter showing further correlation with PBF changes. In conclusion, 2D PC MRI-derived main pulmonary hemodynamic changes reflect changes of RVEF, PBF and 5-month follow-up 6MWD and may be used for future CTEPH patient monitoring after PEA.

## Introduction

Chronic thromboembolic pulmonary hypertension (CTEPH) is a rare, life-threatening disease. Its primary treatment promising cure is pulmonary endarterectomy (PEA) [1–5]. Three-year survival after PEA is 89% compared to only 70% for non-surgical treatment [6].

Following the latest diagnostic algorithm proposed in the”2015 guidelines for the diagnosis and treatment of pulmonary hypertension” of the European Respiratory Society, CTEPH should be diagnosed in specialized centers [2]. Diagnosis includes findings from echocardiography, ventilation/perfusion lung scan, computed tomography pulmonary angiography, right heart catheterization and potentially pulmonary angiography [2]. However, diagnostic approaches are to a certain degree center-specific. Echocardiography, clinical testing such as six-minute walk-distance (6MWD) and right-heart catheterization are usually employed for follow-up.

Magnetic resonance imaging (MRI) has been proposed for CTEPH assessment [7–9]. Regarding the evaluation of pulmonary hypertension and CTEPH in particular, MRI has made distinct advances in the past decade and therefore has become part of our routine perioperative CTEPH patient workup. So far, MRI has shown improved regional and global cardiac function and pulmonary parenchymal perfusion after PEA [7, 10]. In a recent study two-dimensional (2D) phase-contrast (PC) MRI was able to detect hemodynamic changes due to increased pulmonary pressures in CTEPH patients [11]. MRI furthermore offers a choice for radiation-free follow-up.

Thus, the aim of the present study is to identify central pulmonary arterial hemodynamic parameters that reflect clinical, cardiac and pulmonary changes after PEA in CTEPH patients to provide a clinically useful compound marker using 2D PC MRI.

## Materials and methods

### Patient cohort

This retrospective study was approved by the ethics committee of Hannover Medical School (Hannover, Germany, #3408–2016). Written informed consent was obtained from all patients. CTEPH patients undergoing PEA between September 2011 und June 2016 in our hospital were considered for this study. Only patients that completed clinical routine cardiopulmonary MRI before and after surgery were included. MRI is part of our clinical routine workup to evaluate i.a. perfusion defects and cardiac function. Mean pulmonary arterial pressure (mPAP) was documented preoperatively by right heart catheterization and during the postoperative intensive care unit stay before removal of the Swan-Ganz catheter. 6MWD (measured in meters) was obtained in the clinical workup routine before and after PEA. PEA was performed in general anesthesia using cardiopulmonary bypass and deep hypothermia as previously described [12, 13]. A small group of patients received right-heart catherization also after PEA and was considered for subgroup analysis.

### MRI examination

Cardiopulmonary MRI was performed at 1.5T (MAGNETOM Aera and Avanto; Siemens Healthineers GmbH, Erlangen, Germany). An eight-channel torso phased array coil was used for all sequences.

Blood flow was evaluated by retrospectively electrocardiography-gated phase-contrast sequences during free breathing with slices set perpendicular to the main pulmonary artery about 1cm distal of the valve and the bifurcation respectively. The following sequence parameters were used: TR 20ms, TE 2.8ms, flip angle 30°, slice thickness 5mm, matrix 256 × 256, field of view 460mm × 460mm, 75 reconstructed phases, 3 averages, 2 segments, velocity encoding individually adjusted.

Cardiac function was evaluated by retrospectively electrocardiography-gated cine balanced steady-state free precession sequences during short inspiratory breath-holds using short-axis views covering the whole heart with the following sequence parameters: TE 1ms, TR 2.9ms, flip angle 75°, slice thickness 8mm, field of view 290mm x 360mm, matrix size 208 x 256, temporal resolution 35ms, in-plane resolution 1.4mm x 1.4mm, bandwidth/pixel 540Hz/pixel, 30 reconstructed phases.

Regional lung perfusion was evaluated by three-dimensional dynamic contrast enhanced (DCE) time-resolved angiography with stochastic trajectories (TWIST) [14] with the following sequence parameters: TE 0.7ms, TR 2.1ms, flip angle 25°, 40 three dimensional datasets with an update rate of 1.0–1.2s, acquisition matrix 192 x 113, field of view 500mm x 420mm, 0.04mmol/kg Gadoteric acid at 5cc/s intravenously. 30–36 reconstructed coronal slices with a slice thickness of 6mm covering the whole lung were acquired in a single breath-hold.

### Image and data analysis

Phase-contrast and short axis cine MR images were analyzed with cvi42 version 5 (Circle Cardiovascular Imaging Inc., Calgary, Canada). Semiautomatic contour detection with manual adjustment where necessary was applied, a strategy that has previously shown good interobserver agreement [10, 15]. Papillary muscles as well as myocardial trabeculations were included in the blood pool.

**Fig 1:**
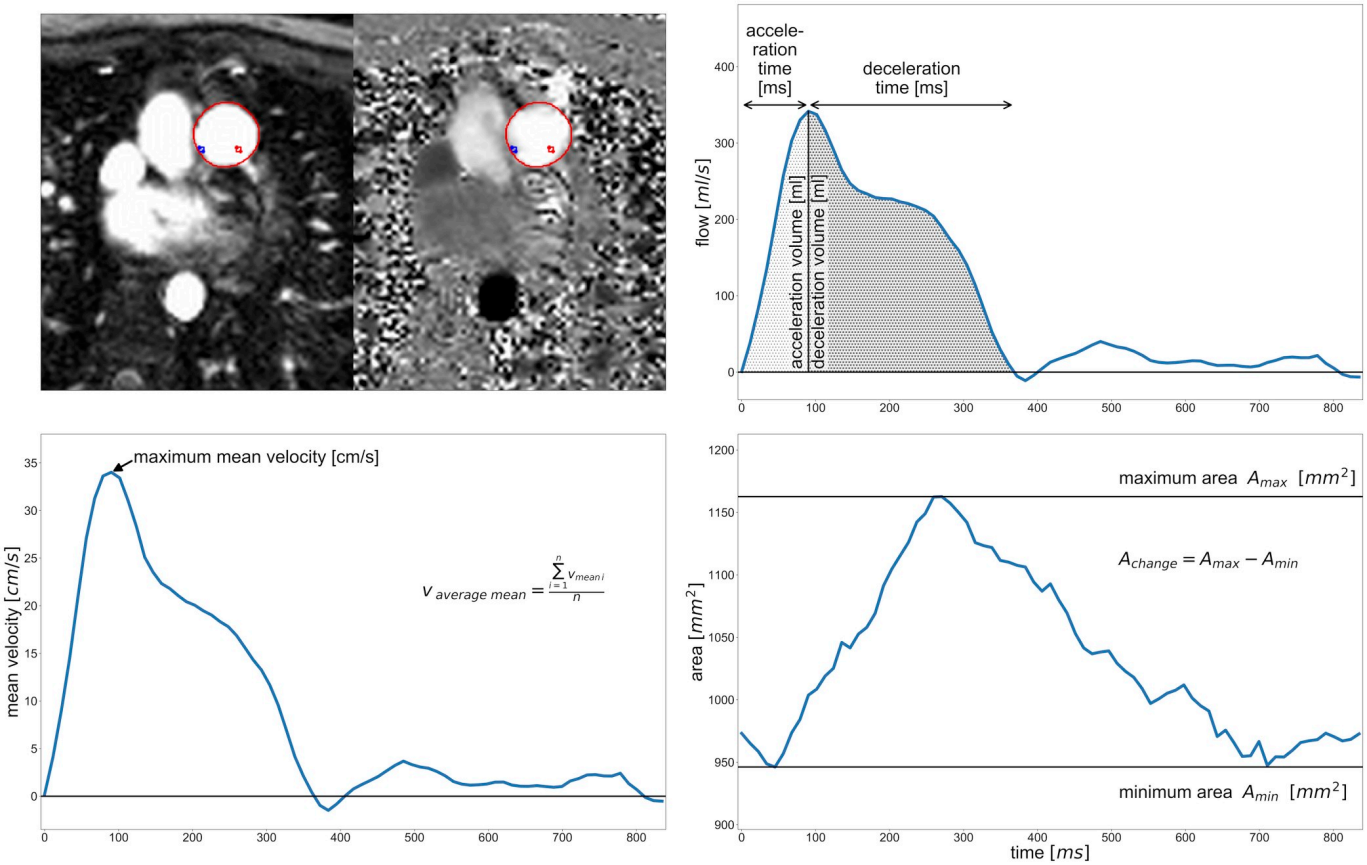
Through-plane two-dimensional phase-contrast measurement in the main pulmonary artery. Illustration of parameters derived from a phase-contrast measurement in the main pulmonary artery as defined by the region of interest in the first image. v_average mean_ = average mean velocity, A_change_ = area change. A_max_ = maximum area, A_min_ = minimum area.

Acceleration time (ms), acceleration volume (ml), deceleration time (ms), deceleration volume (ml), maximum area (mm^2^), minimum area (mm^2^), area change (mm^2^), average mean velocity (cm/s) and maximum mean velocity (cm/s) were determined with phase-contrast cine images. Acceleration time was defined as the time interval from the beginning of systolic antegrade flow up to the time point of maximum antegrade flow. The latter time point was defined as the beginning of deceleration time that ended at the next inflection point of the flow curve except a notch or at the end of antegrade flow. Acceleration volume and deceleration volume were calculated as the integral of flow over acceleration time and deceleration time respectively. Fig 1 illustrates 2D PC MRI parameter acquisition and nomenclature.

Right ventricular (RV) ejection fraction (%, EF), end-diastolic volume (ml, EDV), end-systolic volume (ml, ESV) and myocardial mass (g) were calculated from the short axis cine images.

First-pass DCE MR images were used for calculating parenchymal microvascular pulmonary blood flow (PBF) maps by using pixel-by-pixel deconvolution analysis with PMI-MIKE 0.4 (Platform for Research in Medical Imaging, Leeds, United Kingdom) [16, 17]. The region of interest for calculation of the arterial input function was placed in the main pulmonary artery. PBF maps were segmented after excluding larger pulmonary vessels.

### Statistical analysis

Data are shown as median values with 25^th^ and 75^th^ percentile. Shapiro-Wilk test was applied for normality testing. Data before and after PEA were compared with paired two-sided Wilcoxon rank sum tests as being mainly not normally distributed. One-sided Spearman correlations were applied for rank correlations of parameter ratios before and after PEA.

Subgroup analysis was applied to subjects who also received post PEA right-heart catheterization. Right-heart catheter mPAP, pulmonary vascular resistance and total peripheral resistance were analyzed with Wilcoxon signed-rank tests. Pre/post ratios of these parameters were than correlated to 2D PC MRI parameters *via* one-sided Spearman correlations. The latter 2D PC MRI parameters were restricted to those that showed significance in global (i.e. non-subgroup) analysis before.

Benjamini-Hochberg procedure was applied for control of false discovery rate at level α = 0.05 with adjustment of *p*-values (except for Shapiro-Wilk tests and subgroup analysis).

## Results

**Fig 2:**
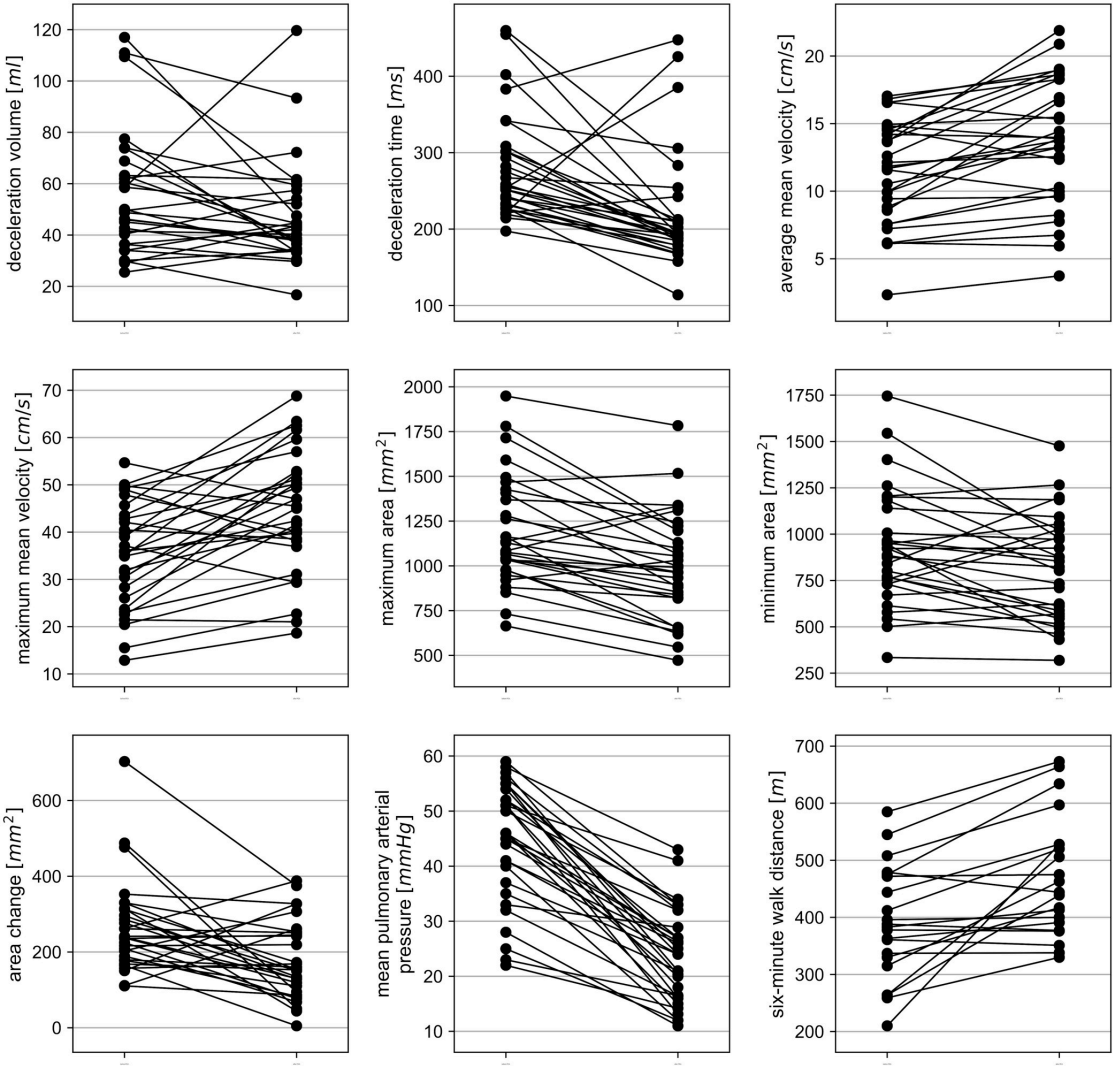
Paired plots of main pulmonary arterial flow and clinical parameters before and after pulmonary endarterectomy. Median values with 25^th^/75^th^ percentiles can be obtained from Table 2.

Between September 2011 und June 2016 we identified 83 CTEPH patients that underwent PEA in our hospital, of which 72 received MRI exams. In total 31 patients with a mean age of 57 years (age range: 20–82 years), 11 women and 20 men, had MRI scans before and after PEA. These were included in this study. Due to a technical error a single 2D PC MRI measurement of the main pulmonary artery before PEA had to be excluded. 6MWD values were available for 22 patients. Table 1 shows the study demographics and examination intervals. The results are summarized in Table 2 and Fig 2.

**Table 1:** Demographics and measurement intervals.

	total	female	male
**number of patients included**	31	11	20
**age [years]**	53 (49; 70)	53 (46; 70)	53 (49; 68)
**body mass index [kg/m**^**2**^**]**	25.4 (23.8; 29.2)	26.8 (24.8; 31.9)	24.6 (23.3; 27.6)
body surface area (Mosteller) [m*m]	2.0 (1.9; 2.2)	1.9 (1.8; 2.1)	2.1 (2.0; 2.2)
**time intervall between preoperative MRI and PEA [days]**	2 (1; 3)	1 (1; 3)	2 (1; 2)
**time intervall between postoperative MRI and PEA [days]**	12 (10; 15)	12 (11; 15)	13 (10; 15)
**time intervall between preoperative 6MWD and PEA [days]**	54 (30; 130)	83 (43; 165)	50 (30; 85)
**time intervall between postoperative 6MWD and PEA [days]**	152 (90; 213)	152 (95; 182)	140 (90; 213)
**time intervall between preoperative mPAP measurement and PEA [days]**	69 (46; 125)	66 (44; 83)	77 (47; 138)
**time intervall between postoperative mPAP measurement and PEA [days]**	2 (1; 3)	2 (1; 3)	2 (1; 3)

This table shows the study population’s demographics and the time intervals between PEA and measurements. All data are shown as median with 25^th^/75^th^ percentile. 6MWD values were available for 22 patients.

**Table 2:** Main pulmonary arterial flow, cardiac and clinical parameters before and after PEA.

parameter	before PEA	after PEA	median change [%]	*p*_*BH*_
**acceleration time [ms]**	100.1 (88.3; 114.3)	125.5 (103.0; 135.5)	25.4	**0.005**
**acceleration volume [ml]**	16.7 (13.2; 21.9)	22.7 (17.4; 30.7)	35.7	**0.001**
**deceleration time [ms]**	254.0 (230.0; 301.5)	193.8 (185.0; 212.4)	-23.7	**<0.001**
**deceleration volume [ml]**	49.1 (37.4; 63.3)	41.2 (35.2; 53.6)	-16.0	**0.038**
**maximum mean velocity [cm/s]**	35.9 (26.6; 43.4)	45.3 (38.3; 52.2)	26.1	**<0.001**
**average mean velocity [cm/s]**	11.8 (8.6; 14.5)	13.9 (10.1; 17.9)	18.2	**<0.001**
**maximum area [mm**^**2**^**]**	1106.2 (991.5; 1398.0)	978.2 (826.9; 1180.4)	-11.6	**<0.001**
**minimum area [mm**^**2**^**]**	904.1 (737.1; 1107.0)	838.7 (574.3; 1014.2)	-7.2	**0.017**
**area change [mm**^**2**^**]**	238.3 (181.8; 308.6)	153.0 (98.6; 249.4)	-35.8	**0.004**
**right ventricular ejection fraction [%]**	35.0 (26.7; 42.5)	48.0 (41.2; 54.4)	37.1	**<0.001**
**right ventricular end-diastolic volume [ml]**	200.0 (150.2; 233.5)	151.0 (126.0; 189.5)	-24.5	**<0.001**
**right ventricular end-systolic volume [ml]**	126.0 (91.0; 163.0)	79.0 (60.5; 101.5)	-37.3	**<0.001**
**right ventricular mass [g]**	89.0 (76.5; 115.0)	79.0 (59.5; 96.5)	-11.2	**<0.001**
**pulmonary blood flow whole lung [ml/min/100ml]**	32.9 (25.2; 51.0)	54.1 (42.0; 69.3)	64.4	**<0.001**
**pulmonary blood flow right lung [ml/min/100ml]**	30.9 (23.9; 49.4)	54.0 (43.3; 69.3)	75.0	**<0.001**
**pulmonary blood flow left lung [ml/min/100ml]**	40.1 (29.6; 49.6)	51.6 (38.1; 64.9)	28.6	**<0.001**
**mean pulmonary arterial pressure [mmHg]**	46.0 (38.5; 53.0)	24.0 (16.5; 28.0)	-47.8	**<0.001**
**six-minute walk distance [m]**	385.5 (331.8; 472.0)	453.5 (393.3; 526.0)	17.6	**0.002**

This table shows the measurements of main pulmonary arterial flow, cardiac and clinical parameters before and after pulmonary endarterectomy as median with 25^th^/75^th^ percentile. The resulting median changes in percent are also reported. The results of Wilcoxon signed rank tests are shown in the last column. p values are corrected via Benjamini-Hochberg method (p_BH_).

After PEA, mPAP decreased significantly by 22 mmHg (*p* = <0.001). In turn 6MWD increased by 68 m (*p* = 0.002). Right heart ejection fraction improved (RVEF, *p*<0.001) and pulmonary parenchymal perfusion increased (PBF, *p*<0.001).

**Fig 3:**
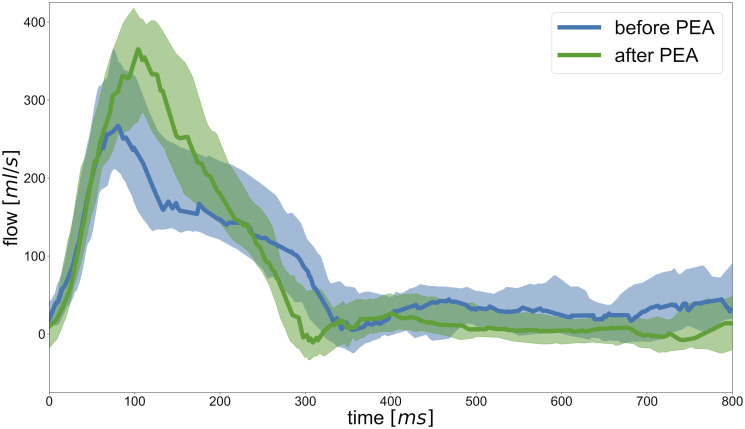
Blood flow curves before and after PEA. Shown are median values with interquartile ranges of all patient’s data interpolated over time.

The ejection phase of flow changed significantly after PEA: acceleration time and volume increased (*p* = 0.005 and *p* = 0.001) while deceleration time and volume decreased (*p*<0.001 and *p* = 0.038). Maximum and average mean velocities significantly increased (*p*<0.001). Fig 3 illustrates blood flow curves before and after PEA.

Significant postoperative decreases could be observed for maximum and minimum area of the main pulmonary artery (*p*<0.001 and *p* = 0.017), as well as area change (*p* = 0.004). RV EDV (*p*<0.001), ESV (*p*<0.001) and mass (*p*<0.001) also decreased significantly.

Spearman correlations of parameter ratios pre/post PEA showed significant correlations of clinical outcome, cardiac function and pulmonary parenchymal perfusion with central pulmonary hemodynamics. Results are shown in Table 3.

**Table 3:** Spearman correlations of main pulmonary arterial flow parameter ratios with clinical, cardiac and perfusion parameter ratios.

	mean pulmonary arterial pressure [mmHg]		pulmonary blood flow whole lung [ml/min/100ml]		right ventricular ejection fraction [%]		six-minute walk distance [m]	
parameter	*p*_*BH*_	rho	*p*_*BH*_	rho	*p*_*BH*_	rho	*p*_*BH*_	*rho*
**acceleration time [ms]**	0.269	-0.21	0.435	-0.07	0.340	-0.15	0.462	0.04
**acceleration volume [ml]**	0.496	0.00	0.362	0.12	0.108	0.34	0.187	0.33
**deceleration time [ms]**	0.435	-0.07	0.196	0.27	0.066	0.38	0.141	0.37
**deceleration volume [ml]**	0.362	0.12	**0.043**	0.44	**0.001**	0.66	**0.010**	0.66
**maximum mean velocity [cm/s]**	0.338	-0.17	0.243	0.24	**0.023**	0.51	**0.030**	0.55
**average mean velocity [cm/s]**	0.444	-0.05	0.155	0.31	**0.043**	0.43	**0.030**	0.55
**maximum area [mm**^**2**^**]**	0.243	0.24	0.362	0.13	0.340	0.15	0.444	0.07
**minimum area [mm**^**2**^**]**	**0.023**	0.50	0.352	0.15	0.471	0.03	0.435	-0.09
**area change [mm**^**2**^**]**	0.295	-0.20	0.338	0.17	0.444	0.05	0.479	0.02

p values are corrected via Benjamini-Hochberg method (p_BH_).

A subgroup of six patients received post PEA right-heart catheterization 293 (236;303) days after PEA. The subgroup consists of 3 men and 3 women with a mean age of 56 years (age range: 43–74; median: 53; 25^th^/75^th^ percentile: 51/61). Right-heart catheter mPAP changed from 47.0 (41.3;56.5) before to 42.0 (25.3;44.8) mmHg after PEA (*p* = 0.12), pulmonary vascular resistance from 8.1 (6.4;11.8) to 6.0 (4.9;6.4) Wood units (*p* = 0.028) and total peripheral resistance 21.0 (17.5;22.6) to 16.1 (14.2;16.4) Wood units (*p* = 0.028). The results of the subgroup parameter ratio correlations are shown in Table 4.

**Table 4:** Spearman correlations of selected main pulmonary arterial flow with right-heart catheterization parameter ratios.

	right-heart catheter mean pulmonary arterial pressure [mmHg]		pulmonary vascular resistance [Wood units]		total peripheral resistance [Wood units]	
parameter	*p*	*rho*	*p*	*rho*	*p*	*rho*
**average mean velocity [cm/s]**	0.133	-0.54	0.394	-0.14	0.272	-0.31
**deceleration volume [ml]**	**0.021**	0.83	0.234	0.37	0.311	0.26
**maximum mean velocity [cm/s]**	0.133	-0.54	0.104	-0.60	0.055	-0.71
**minimum area [mm**^**2**^**]**	0.055	0.71	0.133	0.54	0.198	0.43

Main pulmonary arterial flow parameters are restricted to those that showed significant results in the global (i.e. non-subgroup) analysis.

## Discussion

In this retrospective study we evaluated 2D PC MRI as an integrative method for perioperative evaluation of CTEPH patients treated with PEA. We showed that central pulmonary arterial flow changes assessed with 2D PC MRI correlate with changes of right heart function, pulmonary parenchymal perfusion and clinical parameters. We identified 2D PC MRI-derived deceleration volume ratio as an important parameter that correlates with changes of RVEF, pulmonary parenchymal perfusion 12 days after surgery and changes in 6MWD 5 months after surgery.

Central pulmonary deceleration volume ratio not only interrelated with pulmonary parenchymal perfusion (i.e. PBF) changes, but also with 6MWD and RVEF ratios. These three parameters represent key aspects of successful CTEPH surgery: restoration of lung perfusion with reduction of pulmonary vascular resistance, improvement of right ventricular function and clinical outcome. Thus, deceleration volume ratio represented a non-invasive surrogate compound parameter in this study.

The main purpose of surgical CTEPH treatment is the restoration of lung perfusion through removal of thrombi and scars. As a consequence, we measured significantly increased PBF [10] and main pulmonary arterial blood flow velocities [7, 18, 19]. Central PBF and blood flow velocity ratios did not interrelate significantly. However, whole lung PBF changes showed strong to moderate correlations with deceleration volume ratios. The ratio of acceleration and deceleration time has already been shown to decrease in patients with pulmonary arterial hypertension [20] and to increase in CTEPH patients after PEA [18]. Additionally, acceleration times and volumes have been reported to decrease in patients with pulmonary arterial hypertension [20, 21]. This is in accordance with the presented data, which showed a significant increase after PEA.

PEA reduced right heart strain in our patient cohort. We observed an RVEF increase and in turn RV mass, ESV and EDV decrease [7, 10]. RVEF changes correlated with deceleration volume as well as maximum and average mean velocity ratios. These latter three parameter ratios showed strong to moderate correlations with 6MWD, linking RV function and the clinical 6MWD through main pulmonary arterial hemodynamics. 6MWD, measured 2 months before and 5 months after PEA, increased significantly after PEA. Taking into consideration that MR-derived changes were, in contrast, obtained two days before and 12 days after PEA, data suggests that a perioperative MRI measurement may be capable of anticipating the clinical 5-month follow-up.

MRI has been proposed and continuously validated as perioperative monitoring tool for CTEPH [7, 8, 10, 18, 22]. Inter- and intraobserver variance has been reported previously [10, 20]. 2D PC MRI of the pulmonary trunk is a patient friendly exam, as it can be conducted in free breathing, does not require contrast medium and is fast with around 2 minutes per scan. It allowed the extraction of useful compound markers reflecting the future postoperative clinical status, cardiac function and pulmonary hemodynamics in CTEPH patients. However, the present study is of retrospective nature and limited by the relative small sample size, incomplete follow-up and missing survival data, so that the results may therefore be not perfectly generalizable. Furthermore, 6MWD test was only performed in 22 patients before and after PEA. Nevertheless, we do not assume that the sample size substantially affected the main conclusion of this study. We additionally acknowledge the dependence on postoperative mPAP measurement *via* Swan-Ganz catheter as limitation. Intensive care measurements are less accurate than those obtained from right heart catheterization, but were the only data available for all patients. We, however, do not assume a major impact on the results. We furthermore applied a subgroup analysis to six patients that underwent right-heart catherization after PEA. This subgroup analysis is limited by the small number and a selection bias, as these patients received right heart catheterization due to clinical worsening and suspected recurrent embolism, not as routine clinical follow-up. Despite these limitations, the subgroup analysis additionally showed a strong correlation of deceleration volume with right-heart catheter mPAP ratios.

In conclusion, we showed that hemodynamic changes, assessed by 2D PC MRI in the main pulmonary artery after PEA, reflected changes of right heart function, pulmonary parenchymal perfusion and clinical outcome. We identified deceleration volume ratio as a new parameter and compound marker for perioperative CTEPH patient monitoring.

## Supporting information

S1 Data (XLSX)
